# A global health capstone: an innovative educational approach in a competency-based curriculum for medical students

**DOI:** 10.1186/s12909-020-02070-z

**Published:** 2020-05-19

**Authors:** Stacey Chamberlain, Nicole Gonzalez, Valerie Dobiesz, Marcia Edison, Janet Lin, Stevan Weine

**Affiliations:** 1 University of Illinois Chicago Center for Global Health, 1940 W. Taylor St., 2nd floor, Chicago, IL 60612 USA; 2 Brigham and Women’s Hospital, Harvard Humanitarian Initiative, Harvard Medical School, Boston, MA USA

**Keywords:** Global health, Medical education, Competencies, Capstone, Curriculum

## Abstract

**Background:**

Global health educational programs for medical and public health professionals have grown substantially in recent years. The University of Illinois Chicago College of Medicine (UICOM) began a global medicine (GMED) program for selected students in 2012 and has since graduated four classes. As part of the four-year curriculum, students complete a longitudinal global health capstone project. This paper describes the global health capstone project as an innovative educational tool within a competency-based curriculum.

**Methods:**

The authors define and describe the longitudinal global health capstone including specific requirements, student deliverables, and examples of how the global health capstone may be used as part of a larger curriculum to teach the competency domains identified by the Consortium of Universities for Global Health. The authors also reviewed the final capstone projects for 35 graduates to describe characteristics of capstone projects completed.

**Results:**

The global health capstone was developed as one educational tool within a broader global health curriculum for medical students. Of the 35 capstones, 26 projects involved original research (74%), and 25 involved international travel (71%). Nine projects led to a conference abstract/presentation (26%) while five led to a publication (14%). Twenty-one projects (60%) had subject matter-focused faculty mentorship.

**Conclusions:**

A longitudinal global health capstone is a feasible tool to teach targeted global health competencies and can provide meaningful opportunities for research and career mentorship. Further refinement of the capstone process is needed to strengthen mentorship, and additional assessment methods are needed.

## Background

Participation in global health activities by U.S. medical students has grown substantially in recent decades [1]. Although global health interest has grown, many schools still do not offer structured global health curricula, and there is little standardization for didactic, clinical, scholarly, and cultural components across programs [2, 3]. The past decade saw the development of essential competencies to guide global health curricular development [4–10]. However, many programs lack well-defined competencies outlining critical skills for global health practitioners. The most notable global health competency framework identifies 39 competencies across 11 domains and was published in 2015 by an interdisciplinary expert panel from the Consortium of Universities for Global Health (CUGH). Many of the identified competencies include not only knowledge acquisition but also skills building and attitude formation [4]. Particularly in resource-limited settings involving different cultures, political climates, and power dynamics, effective global health practitioners need competence in cultural humility, inter-professional collaboration, ethical conduct, and promotion of health equity. One major challenge is for educators to identify methods to teach these competencies that will enable students to become successful global health practitioners.

Aspects of various global health curricula have been published. Some describe didactic curricula focused on topics such as cultural competency and communication [11]. Others describe educational formats including e-learning or simulation-based learning to teach competencies such as ethics or professional practice in low-resource settings [12–14]. Many programs involve international electives or service-learning experiences, and best practice approaches have been proposed to help students in short-term global health experiences build skills in cross-cultural effectiveness, capacity building, and collaboration while addressing the needs of host communities and partners [15–18]. Although there are some published descriptions of global health capstones for pharmacy and bioengineering students, there are no known published descriptions of global health capstones as part of an educational curriculum for medical students [19, 20].

The Global Medicine (GMED) Program is a longitudinal four-year track for select medical students that began in 2012, in response to increased interest in global health at the University of Illinois Chicago College of Medicine (UICOM). Completion of a longitudinal capstone project is required as part of the GMED program. Using a global health capstone project as an educational method for medical students is a novel construct. Although capstones are reported in other disciplines, they have not been routinely incorporated into global health medical student programs. Other fields found capstones beneficial because they allow students to:

- Become involved in sustainable impact-oriented research [21].
- Build skills in scholarship and professionalism including writing, presenting, and integrating “core theoretical concepts to form a broad view of professionalism.” [21–23]
- Develop research mentorships and relationships with faculty [21].

In this paper, we describe the global health capstone including how the capstone can be used to teach essential global health competencies, and we report on characteristics of the global health capstone for the first 35 graduates of the GMED Program. This educational method may be of value to other global health educators who wish to develop or strengthen their global health training programs for health professions students.

### Development of the Global Health Capstone

The UICOM GMED Program recruited its first class in 2012 and has since graduated four classes. The program’s goal is to improve the health of populations worldwide by training the next generation of global health leaders [24]. As part of the program, each GMED student must develop, implement, and present a capstone project to successfully complete the program. The global health capstone is defined as a longitudinal scholarly work focused on expanding knowledge and understanding of global health issues among underserved populations throughout the world. The capstone culminates in an oral presentation and reflection paper at the end of the final year of medical school. In 2019, we added an additional requirement of a formal written paper. The capstone is designed to allow students to acquire knowledge and skills through project planning and implementation.

The global health capstone was developed by a multidisciplinary group of faculty with global health and education experience following the steps outlined in the following section:

#### Develop Global Health capstone objectives

Faculty identified global health capstone objectives that focused on skills-building and complemented other components of the global health curriculum. The following objectives were identified for GMED students completing the capstone project:

- Demonstrate and apply an understanding of global health education competencies;
- Identify and utilize credible and scholarly sources of information concerning global health topics and perform an in-depth review of the literature;
- Define an overall purpose and associated specific aims for the project;
- Collaborate with a faculty mentor to ensure adequate progress on the project and receive regular feedback and evaluations;
- Demonstrate effective professional and scientific communication skills through written products and presentations;
- Apply critical thinking skills and a scientific methodology to the analysis of a project.

#### Define capstone focus and parameters

Koplan defines global health as, “an area for study, research, and practice that places a priority on improving health and achieving equity in health for all people worldwide. Global health emphasizes transnational health issues, determinants, and solutions; involves many disciplines within and beyond the health sciences and promotes interdisciplinary collaboration; and is a synthesis of population-based prevention with individual-level clinical care.” [25] Using this definition of global health to frame the focus of the capstone, students were instructed to identify a global health area that they might want to study further.

Because the UICOM has additional special tracks that address urban and rural health, we further required that global health capstone projects should focus on issues in low- and middle-income countries (LMICs) or people from LMICs. This narrower focus allowed our program to avoid overlap with the other programs at our institution that concentrate on domestic health disparities.

#### Describe capstone structure

Capstone projects could vary in structure and content depending on students’ interests. All students received faculty advising that provided guidance for capstone completion. Projects could focus on original global health research or be comprised of curriculum design, program implementation, field practicum, systematic review, or a meta-analysis. All students were expected to demonstrate an understanding of these accepted global health core competencies: [4]

- Global Burden of Disease
- Globalization of Health and Healthcare
- Social and Environmental Determinants of Health
- Capacity Strengthening
- Collaboration, Partnering, and Communication
- Ethics
- Professional Practice
- Health Equity and Social Justice
- Program Management
- Sociocultural and Political Awareness
- Strategic Analysis

Table 1 identifies how the global health capstone can be used as a tool to address each competency domain and provides illustrative examples from completed student projects.

**Table 1:** Global Health Capstones, Competencies, and Examples

CUGH Competency	How Capstones Can Address this Domain	Capstone Example
Global Burden of Disease	Completing literature reviews based on capstone research questions can increase understanding of morbidity and mortality in the population/region of interest and inform about current efforts to address those issues	Systematic review on glaucoma in Africa that included prevalence data, available treatment options, barriers to care, inequalities in access to treatment services, and recommendations for improving care
Globalization of Health and Healthcare	Focusing on how globalization affects health, health systems, and health care delivery by describing different systems of care and their impact on outcomes and expenditures; learning how global trends in health care practice, multinational agreements, and multinational organizations contribute to the quality and availability of health care	Retrospective chart review at a hospital in Kenya to identify patterns of HIV testing services, which were expanded after the Kenya Ministry of Health developed the Kenya AIDS Strategic Framework to scale up HIV testing and counseling services based on recommendations from the World Health Organization; the review identified testing barriers and potential testing biases to make recommendations for expanding testing services
Social and Environmental Determinants of Health	Understanding the social, economic, and environmental factors that impact morbidity, mortality, and access to quality health care services as well as how culture impacts perceptions of health and disease	Retrospective chart review to understand how the West African Ebola epidemic impacted HIV care for soldiers in Sierra Leone; found that the outbreak negatively impacted HIV care for several reasons, including an overburdened health care system and travel restrictions implemented to prevent the spread of Ebola
Capacity Strengthening	Focusing on sharing skills, knowledge, or resources in a manner that will strengthen the programs, infrastructure, or health care workforce available in an area or for a population, particularly when working with local partners and taking an asset-based approach	Key informant interviews with healthcare providers at a teaching hospital in Ghana to understand the hospital’s capacity and needs; informed recommendations for increasing capacity to better serve the hospital’s patient population
Collaboration, Partnering, and Communication	Developing equitable relationships with local partners and key stakeholders to complete projects, working with diverse partners and key stakeholders, and building trust with community partners	Design and delivery of an emergency medicine educational program in collaboration with a hospital in Mongolia; potential for the program to serve as a model for similar training at hospitals throughout the country
Ethics	Completing human subjects research training to gain an understanding of research ethics; applying ethical principles to global health work including managing diverse economic, political, and cultural contexts as well as working with vulnerable populations or in low-resource settings	Evaluation of a global health partnership between a US-based institution and local organization that focused on cervical cancer screening in Senegal; the evaluation outlined strengths and challenges of partnership and made recommendations for creating and maintaining an equitable relationship between partners
Professional Practice	Learning to adapt skill sets, particularly in low-resource settings; developing professionalism through collaborating with a diverse set of partners to implement projects; describing barriers to health and health care in low-resource settings	Needs assessment in Peruvian Amazonian communities in collaboration with local governmental and non-governmental institutions to understand met and unmet health care needs in these remote, rural settings
Health Equity and Social Justice	Using a framework that addresses health inequalities across socially, demographically, or geographically defined populations and engaging marginalized populations in project development and implementation	Survey administered to understand barriers to contraceptive use among adolescents in Nicaragua that found social factors (e.g., fear, lack of communication) played a larger role in non-use compared to physical barriers (e.g., cost)
Program Management	Working through the process of planning, implementing, and/or evaluating global health programs	Survey administered to assess knowledge, preference, and use of HIV prevention methods in high disease prevalence communities in Cape Town, South Africa; involved in managing all phases of the project including drafting a grant proposal and presenting findings
Sociocultural and Political Awareness	Planning a project that considers cultural context and the current local, national, and international landscapes and how those factors impact health	Key informant interviews with health care providers in Ghana to analyze the benefits and challenges of the recently developed National Ambulance Service, how government interventions impacted the health sector, and how factors associated with the patient population (e.g., poverty) impacted care
Strategic Analysis	Using systems thinking to understand the diverse and interrelated factors that impact morbidity, mortality, and access to care and how to consider those complex factors when developing global health programs	Mixed methods community assessment using a community-based participatory research framework with newly settled refugees in Chicago to understand barriers to healthcare access, concerns about accessing care, and how this can be used to improve access

#### Identify capstone requirements and timeline

**Fig. 1:**
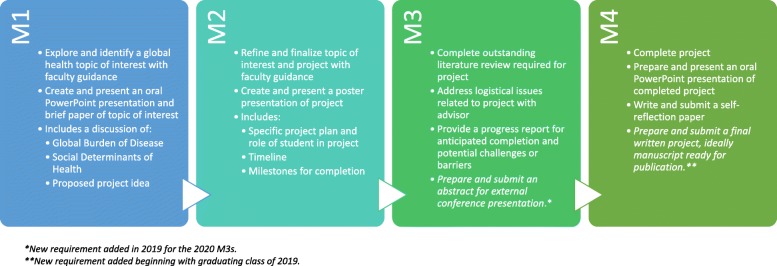
Global Health Capstone Process Map

Specific deliverables were identified for the capstone project that would be required throughout the 4 years of medical school (Fig. 1). During the first year, each student identifies a particular global health issue, performs a literature review, writes a brief paper, and delivers a short oral presentation on his/her selected topic to peers and faculty. In the second year, each student identifies a specific project, defines his/her role in that project, establishes methods and a timeline for project completion, and prepares and presents a scientific poster. In the third and fourth years, students focus on capstone project implementation and evaluation, culminating in oral presentations summarizing their work. In their final presentations, students identify the global health problem addressed; describe the methods, results, and conclusions of the completed projects; and discuss the implications of their projects on the health of underserved communities and on their future practice as global physicians. Graduating students also submit a self-reflection paper upon capstone completion. This paper encourages students to reflect on their accomplishments, articulate the challenges and successes of their projects, and internalize their experiences to translate knowledge acquired to their personal and professional growth.

It is critical to note that the capstone is only one component of the GMED program. In addition to the regular medical school curriculum, the GMED program includes didactic instruction, colloquia, and skills-building workshops described elsewhere [24]. GMED programming also includes exposure to supplementary content (e.g. cultural competency, economic perspectives of global aid, ethics of volunteerism) as well as alternative interactive learning formats including film reviews, book club discussions, and simulation-based cases.

#### Adapt and revise capstone requirements

Based on student feedback and faculty observation, several modifications were made to the original capstone design. We revised and more precisely defined the focus for global health capstone projects; this adaptation was made in response to project proposals that did not clearly have transnational health relevance. We adapted the capstone objectives to include updated global health competencies. The original GMED curriculum addressed global health competencies identified in 2010 by the Global Health Education Consortium. An expanded and updated list of competencies was identified by CUGH in 2018, and we revised our capstone objectives and guidelines to reflect this change [4, 10]. A written scholarly paper was added as a requirement for 2019 graduates. Submitting an abstract to a non-UICOM conference was added as a third year requirement that will take effect starting in 2020. Ongoing capstone adaptations based on the findings of this review include instituting a new mentorship program (see Discussion).

## Methods

We performed a retrospective review of graduating medical student capstone projects from the first three GMED cohorts (2016–2018) to determine the nature and range of projects completed. We were specifically interested in: [1] the types of projects completed [2]; whether the students identified a project faculty mentor [3]; whether the student travelled internationally as part of the project [4]; whether the project was related to the student’s chosen residency specialty; and [5] whether the project led to formal scholarship including an abstract/poster presentation or publication. To make these determinations, two authors (S.C., V.D., M.E., or J.L.) completed an independent document review of the capstone PowerPoint presentations of the first 35 graduating students. The authors then met, and any areas of disagreement were discussed until consensus was reached.

The criteria for each of the interest areas above are described here. For “types of projects completed,” we used the broad categories of clinical research, education, quality improvement, and service similar to other analyses [26]. We further characterized non-interventional clinical research capstones as systematic reviews versus original research. For original research projects, we determined whether projects used quantitative, qualitative, or mixed-methods. This information was gathered as part of our iterative process for the global health capstone design to help inform our team of potential areas of curricular enhancement. For example, if many students were completing qualitative analyses, we could consider adding additional qualitative assessment instruction as part of the formal GMED curriculum.

As noted previously, all students were assigned a faculty advisor who provided guidance throughout the capstone process. However, these advisors were not necessarily project mentors who possessed content expertise in the capstone area of focus, or may not have directly worked with the students on specific projects. Students who had capstone project-specific mentors explicitly mentioned in their presentations were considered to have had “project faculty mentors.”

It was determined that the student travelled internationally based on the final presentation. Similarly, the extent to which the project was related to residency specialty was determined by the judgment of the faculty reviewers, who decided if there was an evident relationship between the subject matter of the capstone project and the known scope of practice and subject matter relevance to a medical specialty. For example, a project on post-partum hemorrhage for a student who matched into orthopedic surgery was determined to be “unrelated,” but a project looking at point-of-care ultrasound use in emergency departments for a student who matched into emergency medicine was determined to be “related.”

A project led to a scholarly abstract/poster presentation and/or publication if students identified this in their presentations or if a PubMed search done at the time of our study revealed it. Publications of capstone work that occurred after students graduated, identified by the PubMed search, were included in this report.

Finally, one author (N.G.) identified capstone projects that illustrated aspects of the CUGH competencies based on student final presentations (Table 1).

## Results

The capstone is designed to enhance students’ scholarly skills and knowledge. As noted, students were given some flexibility as to the capstone structure and format. Of the initial 35 program graduates, 32 (91%) completed capstones involving non-interventional clinical research. Of those, five were systematic reviews, and one was a case series. Twenty-six capstones were categorized as original research. Of those, ten (39%) used mixed methods, ten (39%) used quantitative methods, and six (23%) used qualitative methods. Of the capstones that were not clinical research, two were education-focused and involved curriculum development, and one was a quality improvement project.

While all students had faculty advisors, 21 capstones (60%) involved projects where students had additional dedicated faculty mentorship, meaning they worked with a faculty member who possessed subject matter expertise and guided their capstone development and implementation. The remaining 14 projects (40%) were implemented in a more independent manner.

Capstones included projects in 14 different countries; eight additional projects had a transnational global health focus, and four projects focused on domestic and/or refugee populations in the U.S. (Table 2). Twenty-five capstones (71%) involved an international field experience.

**Table 2:** Capstone Locations

Countries (# of Projects Completed)	
Colombia (1)	
Mexico (1)	
New Zealand (1)	
Nicaragua (1)	
Palestine (1)	
Peru (1)	
Senegal (1)	
Sierra Leone (1)	
Uganda (1)	
Dominican Republic (2)	
Mongolia (2)	
Kenya (3)	
South Africa (3)	
Ghana (4)	
United States (4)	

Multiple medical specialty areas were identified, with the largest percentage of projects focused on emergency medicine (29%), obstetrics/gynecology (17%), and primary care (14%). Other projects focused on internal medicine (9%), psychiatry (6%), neurology (3%), ophthalmology (3%), and pediatrics (3%). In addition, six projects (17%) did not clearly align with a medical specialty area and instead focused on topics including environmental health, medical ethics, health systems, medical education, and mobile health (mHealth) smartphone applications. Eighteen students (51%) completed capstones related to their chosen medical residency specialty. Twenty-six percent of students presented capstone-related abstracts or presentations at conferences, and five (14%) authored peer-reviewed publications related to their capstones.

## Discussion

### A longitudinal global health capstone is feasible for medical students

Overall, we found that a four-year longitudinal capstone is feasible. Skill development, knowledge acquisition, and mentorship were among the most important outcomes of the capstone process, and those outcomes were not dependent on students completing a single long-term project. Although many students had more than one specific project during their capstone, all students went through the same four-year longitudinal process with defined deliverables during each year of medical school. We found the focus on process important to provide a continuum of mentorship and opportunity to build cross-disciplinary skills, while allowing the students flexibility to change their specific final project focus and adapt to barriers they encountered in project implementation.

Giving students the flexibility to change their final project focus over time enables students to pursue meaningful scholarship related to their future specialties as their career interests evolve. In addition, it allows some students to participate in different aspects of serial short-term projects. One of the greatest challenges for students we noted was in identifying projects; this may be mitigated by directing students to focus on building translatable skills rather than focusing on specific geographic project locations, patient populations, or narrow topical areas.

We observed personal and professional growth of students as they faced challenges in project planning and implementation. The obstacles confronted by our students reflect real world challenges of global health work and provided student learning opportunities. A longitudinal 4-year capstone with defined progressive requirements exposes students to the challenges of global health work including mentor identification, ethical review of human subjects research, data collection delays, and lack of student availability at times due to competing priorities of exams and clerkships.

### Capstones create an opportunity for dedicated mentorship

Rather than assigning project mentors, students are encouraged to pursue global health capstone projects with mentors they align with. Although every student is assigned an advisor to provide support for program completion, these advisors are not necessarily content experts in the student’s research area of interest. Sixty percent of GMED graduates ultimately completed a capstone project where they received dedicated topic-specific faculty mentorship. Completion of quality global health capstones could be enhanced with strategic efforts to create more structured mentorship and recruit more global health faculty.

The mentorship process for successful capstone development and completion can be improved by making sure that every student identifies a research mentor. We anticipate that dedicated mentors can improve the quality of the capstone experience and help the students create a stronger final scholarly product. We found that 26% of students presented capstone-related abstracts at conferences, and 14% were able to publish work related to their capstones. With dedicated project mentorship for every student, we aim to increase the number of students producing quality global health scholarship. For 2020, we added a requirement that students must submit a global health abstract to an external conference in the third year, and in 2019 we added the requirement that students submit a final written scholarly paper in addition to the oral presentations that were part of the original capstone requirements.

An additional aim of expanding our pool of capstone mentors is to increase multidisciplinary mentorship and collaboration among more varied medical specialty areas. When the program was founded, emergency medicine had strong representation among GMED program faculty, which may explain why almost a third of student capstones were in that specialty area. We have implemented a new structured mentorship program that provides wider faculty representation to ensure that students are provided necessary support and guidance regardless of the students’ chosen area of interest.

### The capstone is synergistic with other modalities for teaching CUGH competencies

The global health capstone addresses, in part, each competency domain identified by CUGH, but the global health capstone is part of a larger curriculum that employs multiple educational modalities. Some CUGH competencies may be better achieved through these alternate methods, such as lectures, group discussion, and simulation-based exercises. We have also added additional didactic content to support student capstone success and competency attainment including skills-based workshops that focus on community engagement, global health research and scholarship, as well as global health policy and advocacy.

Many students were able to complete global health capstones that did not require international travel. Considering personal and financial restrictions that may affect students’ ability to travel, the global health capstone reinforces the view that global health can focus on transnational health issues addressing health equity, and one need not always travel to participate in effective global health work.

#### Limitations

This paper aimed to provide a description of the global health capstone including types of projects completed; however, it did not identify clear metrics for capstone success or evaluate student capstone projects. It identified how capstones may be used to teach global health competency domains but did not determine the effectiveness of this approach nor if there are particular domains that are better addressed by this educational tool. Finally, numerous challenges in the assessment of global health competencies have been identified [27, 28]. Attempts have been made to develop measures such as surveys, structured instruments, and self-assessments in order to objectively assess global health competencies, but more research is needed in this area, including developing validated measures to assess global health capstones [12, 29, 30].

## Conclusions

As the bar is raised on global health education beyond just international electives, students need integrated and formalized programming that enables them to develop skills and the ability to apply concepts in impactful global health endeavors. A structured global health capstone is one method for teaching global health competencies and preparing students for careers as global health practitioners and leaders. The implementation of a global health capstone in medical school is feasible and shows promise as an educational tool that may help teach essential global health core competencies as part of a broader curriculum. Well-defined criteria and expectations for global health capstones may improve scholarly quality and productivity, and strong mentorship is essential for successful capstone and program completion. Further refinement of the global health capstone may allow educators to help students build scholarly skills and target additional competency domains.

## Data Availability

The datasets used and/or analysed during the current study are available from the corresponding author on reasonable request.

## Abbreviations

GMED: Global Medicine Program
UICOM: University of Illinois College of Medicine
CUGH: Consortium of Universities for Global Health
LMIC: Low- and Middle-Income Country
HIV: Human Immunodeficiency Virus
AIDS: Acquired Immunodeficiency Syndrome
mHealth: mobile health
